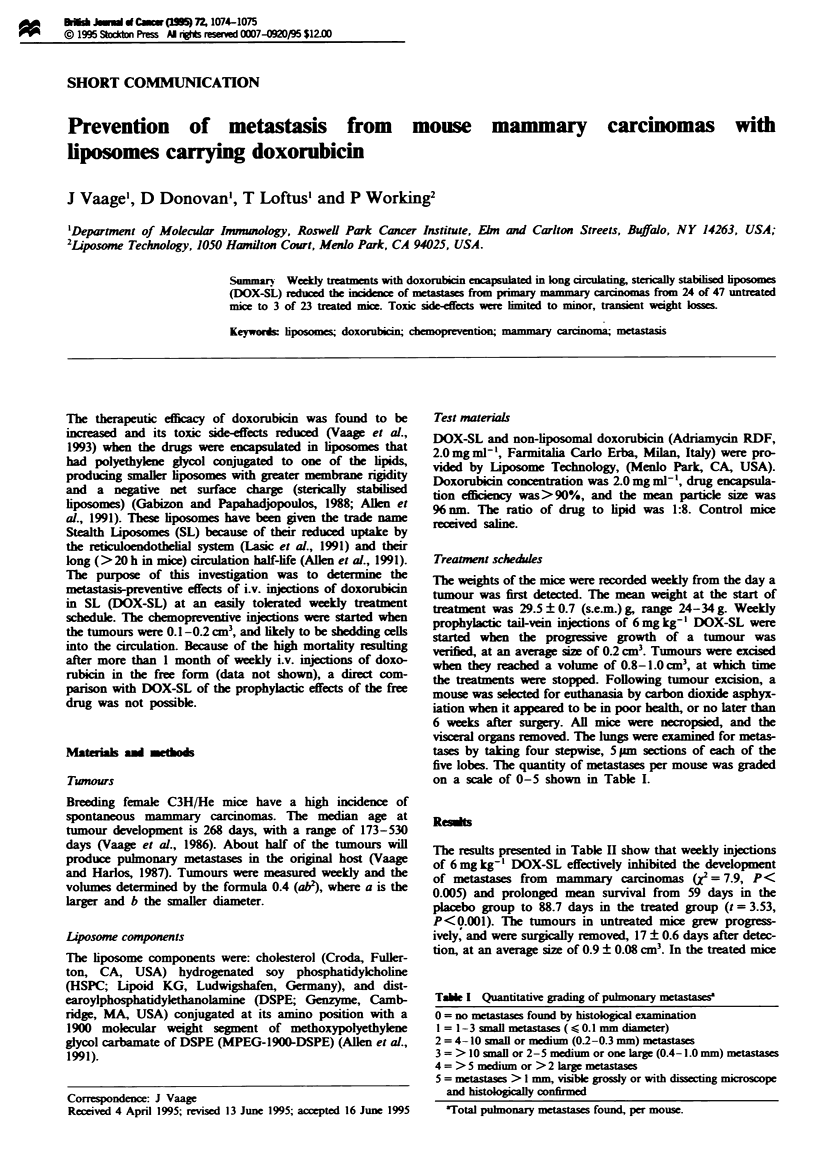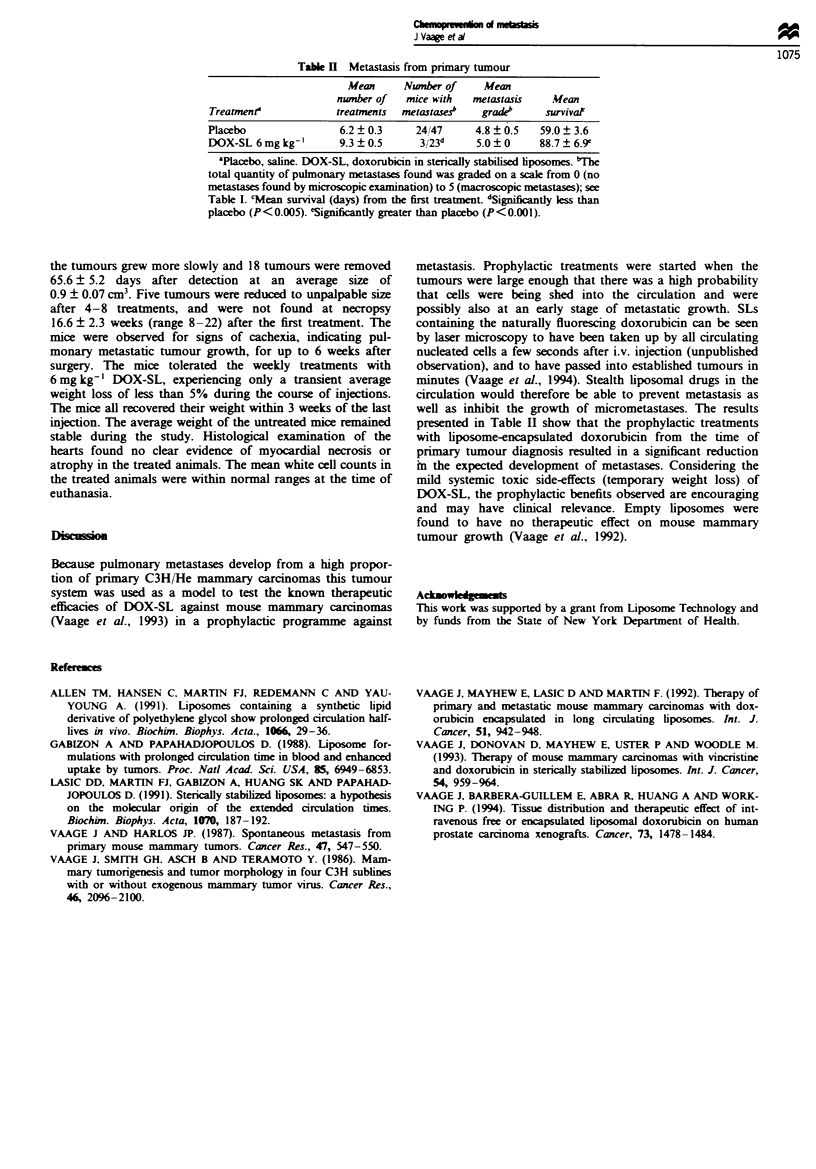# Prevention of metastasis from mouse mammary carcinomas with liposomes carrying doxorubicin.

**DOI:** 10.1038/bjc.1995.467

**Published:** 1995-11

**Authors:** J. Vaage, D. Donovan, T. Loftus, P. Working

**Affiliations:** Department of Molecular Immunology, Roswell Park Cancer Institute, Buffalo, NY 14263, USA.

## Abstract

Weekly treatments with doxorubicin encapsulated in long circulating, sterically stabilised liposomes (DOX-SL) reduced the incidence of metastases from primary mammary carcinoma from 24 of 47 untreated mice to 3 of 23 treated mice. Toxic side-effects were limited to minor, transient weight losses.


SHORT COMMUNICATION

### Prevention of metastasis from mouse mammary carcinomas with liposomes carrying doxorubicin

J Vaage[1], D Donovan[1], T Loftus[1] and P Working[2]

[1]*Department of Molecular Immunology, Roswell Park Cancer Institute, Elm and Carlton Streets, Buffalo, NY 14263, USA;* [2]*Liposome Technology, 1050 Hamilton Court, Menlo Park, CA 94025, USA.*

**Summary** Weekly treatments with doxorubicin encapsulated in long circulating, sterically stabilised liposomes (DOX-SL) reduced the incidence of metastases from primary mammary carcinomas from 24 of 47 untreated mice to 3 of 23 treated mice. Toxic side-effects were limited to minor, transient weight losses.

**Keywords:** liposomes; doxorubicin; chemoprevention; mammary carcinoma; metastasis

The therapeutic efficacy of doxorubicin was found to be increased and its toxic side-effects reduced (Vaage *et al.*, 1993) when the drugs were encapsulated in liposomes that had polyethylene glycol conjugated to one of the lipids, producing smaller liposomes with greater membrane rigidity and a negative net surface charge (sterically stabilised liposomes) (Gabizon and Papahadjopoulos, 1988; Allen *et al.*, 1991). These liposomes have been given the trade name Stealth Liposomes (SL) because of their reduced uptake by the reticuloendothelial system (Lasic *et al.*, 1991) and their long (>20 h in mice) circulation half-life (Allen *et al.*, 1991). The purpose of this investigation was to determine the metastasis-preventive effects of i.v. injections of doxorubicin in SL (DOX-SL) at an easily tolerated weekly treatment schedule. The chemopreventive injections were started when the tumours were 0.1–0.2 $cm^3$, and likely to be shedding cells into the circulation. Because of the high mortality resulting after more than 1 month of weekly i.v. injections of doxorubicin in the free form (data not shown), a direct comparison with DOX-SL of the prophylactic effects of the free drug was not possible.

### Materials and methods

### *Tumours*

Breeding female C3H/He mice have a high incidence of spontaneous mammary carcinomas. The median age at tumour development is 268 days, with a range of 173–530 days (Vaage *et al.*, 1986). About half of the tumours will produce pulmonary metastases in the original host (Vaage and Harlos, 1987). Tumours were measured weekly and the volumes determined by the formula 0.4 ($ab^2$), where $a$ is the larger and $b$ the smaller diameter.

### *Liposome components*

The liposome components were: cholesterol (Croda, Fullerton, CA, USA) hydrogenated soy phosphatidylcholine (HSPC; Lipoid KG, Ludwigshafen, Germany), and distearoylphosphatidylethanolamine (DSPE; Genzyme, Cambridge, MA, USA) conjugated at its amino position with a 1900 molecular weight segment of methoxypolyethylene glycol carbamate of DSPE (MPEG-1900-DSPE) (Allen *et al.*, 1991).

Correspondence: J Vaage
Received 4 April 1995; revised 13 June 1995; accepted 16 June 1995

### *Test materials*

DOX-SL and non-liposomal doxorubicin (Adriamycin RDF, 2.0 mg $ml^{-1}$, Farmitalia Carlo Erba, Milan, Italy) were provided by Liposome Technology, (Menlo Park, CA, USA). Doxorubicin concentration was 2.0 mg $ml^{-1}$, drug encapsulation efficiency was >90%, and the mean particle size was 96 nm. The ratio of drug to lipid was 1:8. Control mice received saline.

### *Treatment schedules*

The weights of the mice were recorded weekly from the day a tumour was first detected. The mean weight at the start of treatment was 29.5 ± 0.7 (s.e.m.) g, range 24–34 g. Weekly prophylactic tail-vein injections of 6 mg $kg^{-1}$ DOX-SL were started when the progressive growth of a tumour was verified, at an average size of 0.2 $cm^3$. Tumours were excised when they reached a volume of 0.8–1.0 $cm^3$, at which time the treatments were stopped. Following tumour excision, a mouse was selected for euthanasia by carbon dioxide asphyxiation when it appeared to be in poor health, or no later than 6 weeks after surgery. All mice were necropsied, and the visceral organs removed. The lungs were examined for metastases by taking four stepwise, 5 μm sections of each of the five lobes. The quantity of metastases per mouse was graded on a scale of 0–5 shown in Table I.

### Results

The results presented in Table II show that weekly injections of 6 mg $kg^{-1}$ DOX-SL effectively inhibited the development of metastases from mammary carcinomas ($\chi^2 = 7.9$, $P <$ 0.005) and prolonged mean survival from 59 days in the placebo group to 88.7 days in the treated group ($t = 3.53$, $P < 0.001$). The tumours in untreated mice grew progressively, and were surgically removed, 17 ± 0.6 days after detection, at an average size of 0.9 ± 0.08 $cm^3$. In the treated mice

**Table I** Quantitative grading of pulmonary metastases[a]

| |
|---|
| 0 = no metastases found by histological examination |
| 1 = 1–3 small metastases (⩽ 0.1 mm diameter) |
| 2 = 4–10 small or medium (0.2–0.3 mm) metastases |
| 3 = > 10 small or 2–5 medium or one large (0.4–1.0 mm) metastases |
| 4 = > 5 medium or > 2 large metastases |
| 5 = metastases > 1 mm, visible grossly or with dissecting microscope and histologically confirmed |

[a]Total pulmonary metastases found, per mouse.

Table II Metastasis from primary tumour

| Treatment[a] | Mean number of treatments | Number of mice with metastases[b] | Mean metastasis grade[b] | Mean survival[c] |
|---|---|---|---|---|
| Placebo | 6.2 ± 0.3 | 24/47 | 4.8 ± 0.5 | 59.0 ± 3.6 |
| DOX-SL 6 mg kg$^{-1}$ | 9.3 ± 0.5 | 3/23[d] | 5.0 ± 0 | 88.7 ± 6.9[e] |

[a]Placebo, saline. DOX-SL, doxorubicin in sterically stabilised liposomes. [b]The total quantity of pulmonary metastases found was graded on a scale from 0 (no metastases found by microscopic examination) to 5 (macroscopic metastases); see Table I. [c]Mean survival (days) from the first treatment. [d]Significantly less than placebo ($P<0.005$). [e]Significantly greater than placebo ($P<0.001$).

the tumours grew more slowly and 18 tumours were removed 65.6 ± 5.2 days after detection at an average size of 0.9 ± 0.07 cm$^3$. Five tumours were reduced to unpalpable size after 4–8 treatments, and were not found at necropsy 16.6 ± 2.3 weeks (range 8–22) after the first treatment. The mice were observed for signs of cachexia, indicating pulmonary metastatic tumour growth, for up to 6 weeks after surgery. The mice tolerated the weekly treatments with 6 mg kg$^{-1}$ DOX-SL, experiencing only a transient average weight loss of less than 5% during the course of injections. The mice all recovered their weight within 3 weeks of the last injection. The average weight of the untreated mice remained stable during the study. Histological examination of the hearts found no clear evidence of myocardial necrosis or atrophy in the treated animals. The mean white cell counts in the treated animals were within normal ranges at the time of euthanasia.

### Discussion

Because pulmonary metastases develop from a high proportion of primary C3H/He mammary carcinomas this tumour system was used as a model to test the known therapeutic efficacies of DOX-SL against mouse mammary carcinomas (Vaage *et al.*, 1993) in a prophylactic programme against metastasis. Prophylactic treatments were started when the tumours were large enough that there was a high probability that cells were being shed into the circulation and were possibly also at an early stage of metastatic growth. SLs containing the naturally fluorescing doxorubicin can be seen by laser microscopy to have been taken up by all circulating nucleated cells a few seconds after i.v. injection (unpublished observation), and to have passed into established tumours in minutes (Vaage *et al.*, 1994). Stealth liposomal drugs in the circulation would therefore be able to prevent metastasis as well as inhibit the growth of micrometastases. The results presented in Table II show that the prophylactic treatments with liposome-encapsulated doxorubicin from the time of primary tumour diagnosis resulted in a significant reduction in the expected development of metastases. Considering the mild systemic toxic side-effects (temporary weight loss) of DOX-SL, the prophylactic benefits observed are encouraging and may have clinical relevance. Empty liposomes were found to have no therapeutic effect on mouse mammary tumour growth (Vaage *et al.*, 1992).

### Acknowledgements

This work was supported by a grant from Liposome Technology and by funds from the State of New York Department of Health.

### References

ALLEN TM, HANSEN C, MARTIN FJ, REDEMANN C AND YAU-YOUNG A. (1991). Liposomes containing a synthetic lipid derivative of polyethylene glycol show prolonged circulation half-lives *in vivo*. *Biochim. Biophys. Acta.*, **1066**, 29–36.

GABIZON A AND PAPAHADJOPOULOS D. (1988). Liposome formulations with prolonged circulation time in blood and enhanced uptake by tumors. *Proc. Natl Acad. Sci. USA*, **85**, 6949–6853.

LASIC DD, MARTIN FJ, GABIZON A, HUANG SK AND PAPAHADJOPOULOS D. (1991). Sterically stabilized liposomes: a hypothesis on the molecular origin of the extended circulation times. *Biochim. Biophys. Acta*, **1070**, 187–192.

VAAGE J AND HARLOS JP. (1987). Spontaneous metastasis from primary mouse mammary tumors. *Cancer Res.*, **47**, 547–550.

VAAGE J, SMITH GH, ASCH B AND TERAMOTO Y. (1986). Mammary tumorigenesis and tumor morphology in four C3H sublines with or without exogenous mammary tumor virus. *Cancer Res.*, **46**, 2096–2100.

VAAGE J, MAYHEW E, LASIC D AND MARTIN F. (1992). Therapy of primary and metastatic mouse mammary carcinomas with doxorubicin encapsulated in long circulating liposomes. *Int. J. Cancer*, **51**, 942–948.

VAAGE J, DONOVAN D, MAYHEW E, USTER P AND WOODLE M. (1993). Therapy of mouse mammary carcinomas with vincristine and doxorubicin in sterically stabilized liposomes. *Int. J. Cancer*, **54**, 959–964.

VAAGE J, BARBERA-GUILLEM E, ABRA R, HUANG A AND WORKING P. (1994). Tissue distribution and therapeutic effect of intravenous free or encapsulated liposomal doxorubicin on human prostate carcinoma xenografts. *Cancer*, **73**, 1478–1484.